# Basic steps in establishing effective small group teaching sessions in medical schools

**DOI:** 10.12669/pjms.294.3609

**Published:** 2013

**Authors:** Sultan Ayoub Meo

**Affiliations:** 1 Sultan Ayoub Meo, MBBS, PhD, FRCP, Department of Physiology, College of Medicine, King Saud University, Riyadh, Saudi Arabia.

**Keywords:** Small group teaching, Learning, Medical Schools

## Abstract

Small-group teaching and learning has achieved an admirable position in medical education and has become more popular as a means of encouraging the students in their studies and enhance the process of deep learning. The main characteristics of small group teaching are active involvement of the learners in entire learning cycle and well defined task orientation with achievable specific aims and objectives in a given time period. The essential components in the development of an ideal small group teaching and learning sessions are preliminary considerations at departmental and institutional level including educational strategies, group composition, physical environment, existing resources, diagnosis of the needs, formulation of the objectives and suitable teaching outline. Small group teaching increases the student interest, teamwork ability, retention of knowledge and skills, enhance transfer of concepts to innovative issues, and improve the self-directed learning. It develops self-motivation, investigating the issues, allows the student to test their thinking and higher-order activities. It also facilitates an adult style of learning, acceptance of personal responsibility for own progress. Moreover, it enhances student-faculty and peer-peer interaction, improves communication skills and provides opportunity to share the responsibility and clarify the points of bafflement.

## INTRODUCTION

Over the last four decades, small-group teaching and learning has achieved an admirable position in medical education and is well-liked as a means of encouraging students and enhances the process of deep learning. Small group learning is defined as a process of learning that takes place when students work together in groups of 8-10.^1^^,^^2^ Small group teaching and learning sessions increase student interest, retention of knowledge, enhance transfer of concepts to novel issues, students' critical skills, teamwork ability, self-directed learning, communication skills, student-faculty and peer-peer interaction.^3^^,^^4^ It provides opportunity for articulating thoughts and fomenting the views. There are greater expectations of the graduates' ability to communicate with patients and at different academic as well as scientific floors. Small group discussion provides chance to the students to monitor their own learning and thus gain an experience of self-direction and independence of the instructors.^5^

***Characteristics of small group teaching:*** The most important characteristics of small group teaching are the active involvement of learners in the entire learning cycle, well defined task orientation with achievable specific aims and objectives in a given time and the reflection based on the experience and deep learning.^1^

***The advantages of small group teaching:*** Small group teaching has many advantages to offer the learner. These includes self direction and active learning, encourages reflection upon and control of learning activities and development of self-regulatory skills conducive to lifelong learning. It develops self-motivation, investigating of issues, allows the student to test their thinking, hypothesis, deep learning and higher-order activities such as analysis, evaluation and synthesis. It facilitates an adult style of learning, acceptance of personal responsibility for own progress. It promotes transferable skills such as leadership, teamwork, organization, prioritization, and encouragement to others, problem solving, and time management skills.^6^^-^^8^

***Small group teaching methods:*** There is range of small group teaching methods depending upon the expected learning outcomes. These methods are adopted in different medical schools according to their curriculum, resources and academic environment. The important small group methods popular in medical schools are tutorials, seminar, problem based learning, bed side and ward based clinical teaching.^8^ This is a commonly used technique, relies on the teacher developing stimulus questions on which the group should work before discussing and sharing their findings. The design of small group teaching sessions should be based on the major principles including the introduction of the topic, ground rules, group maintenance role and the task role, activity, briefing and debriefing.^9^ The Tuckman's group development model^10^ is important to understand and establish the small group teaching sessions.

**Table-I:** The Tuckman's group development model stages^10^

**Forming: **The members of the team know each other, exchange information, understand how distribute the task in small group teaching sessions and how they respond.
**Storming: **Addresses the issues such as what problems are supposed to solve, how they will function independently and together and what role they will accept.
**Norming: **Manages to have specific aim and objectives and a mutual plan. Give up own ideas and agree with others in order to make the team function. The members take the responsibility and have the ambition to work for the success of the team's goals.
**Performing: **The team functions as a unit as they find ways to perform the task smoothly and effectively without inappropriate conflict. The team members are able to handle the decision-making process.^10^^,11^

**Table-II:** Basic steps in the development of effective small group teaching sessions

Preliminary considerations at Department / Institutional level
Check and utilize all available resources
Establish educational strategies
Check the availability of appropriate place for small group teaching sessions
Establish group Size and group composition
Appropriate physical environment
Suitable small group teaching climate
Diagnosis of the needs
Formulation of the objectives
Develop small group teaching outlines
Group maintenance role
The task role
Completing the session

***[i] Preliminary considerations: ***A departmental planning committee for small group teaching sessions should be established to develop and coordinate the implementation of the sessions. All the members of the committee should be assigned clear established roles and responsibilities. It should be ensured that enough well trained faculty members / staff are available to facilitate the groups. The chairperson should ensure that Committee meetings promote a positive, collaborative working environment. Committee members conceive problems, create and weigh likely alternative solutions, envision the probable results of each alternative and select the best course of action.^12^ The decisions should be based on how best to facilitate the desired outcome. The desired outcomes should be selected first and the sessions should be developed to support the intended outcome. The instructions must be clear in that, student have information seeking skills such as the ability to use the Electronic Spirometer as an outcome of lung function lab instruction. Performance-based approach can be applied including what sort of physicians will be produced. The educational outcomes can be clearly and unambiguously specified. It encourages the teacher and the student to share responsibility, and guides student assessment and course evaluation. Before the start of the session, availability of appropriate resources should be ensured, faculty and comprehensive plan should be approved by the department. Moreover, in the beginning, introductory session can be arranged to develop frank, constructive and direct relationship between teacher and students. Pleasant environment promote a culture through knowing, valuing student & respect for individuals. In pleasant environment student participate more freely and feel safe.

***[ii] Knowledge-skill and attitude required by the tutor and students:*** The tutor and students needs a particular skill, knowledge and attitude in small group teaching. The tutor must have command on the specific skills such as listening, responding, questioning, higher order questions, setting clear goals, and handling quieter, dominant, brilliant and poor students. Moreover, the tutor must understand the learning styles of the students and accept the ideology of change in attitude to moving from teacher-centred to student-centred teaching. Similarly, students also acquire verbal, communication, confidence / self esteem skills and accept change in attitude towards accepting the challenge in small group teaching instead of that only the teacher can discuss, explain and reply because teacher is expert.^6^

***[iii] Educational strategies:*** In the small group sessions a scientific approach can be adopted to help and promote the learning in context, so that the higher-level learning objectives can easily be achieved. Besides, it may facilitate the students to apply their knowledge in the basic sciences to the clinical situation when learning is centered on a clinical problem in an integrated approach. Irrelevant information can be removed from the session, particularly if teaching and learning were focused around clinical scenarios. Schedule about contents, teaching faculty, exact date and time, place of small group session should be provided to all the students one week before the commencement of small group sessions.

***[iv] Availability of rooms for small group teaching sessions:*** The departmental committee should ensure about appropriate rooms for the small group sessions. The room should allow the group to work in a circle allowing all participants to see and hear each other. Make sure the room is suitable and not overcrowded, is well arranged, lighted and has appropriate visual aids if required, such as flip chart, overhead projector, chalkboard and PowerPoint.

***[v] Group Size:*** In small group teaching the size of the group plays a significant role in the mechanism of teaching and learning. The smaller the group the greater is the likelihood of close relationships, full participation, and consonance of aims and objectives. However, while designing a group size, keep in mind that, larger group may impair the flavor of small group teaching and learning. Each group may consist of 8-10 students and a faculty member must be assigned for each group.

***[vi] Group Composition:*** As a general rule, a mixture of students in each group provides the best chemistry for interaction and achievement of small group teaching and learning tasks. Such qualities as age, gender, nationality, and personality should be taken into account. Considering the significance of cognitive learning with problem solving, the mixing of intelligent and enthusiastic students with their slower counterparts can facilitate the learning process between the students. Therefore, it is suggested to arrange the each group based on age, gender, ethnicity, and their previous academic achievements / record.

***[vii] Physical Environment:*** Physical environment has an important role in the small group teaching sessions. There are quite few imperative factors in group dynamics, communication, perception of status, emergence of leadership which is affected by different factors including the physical position of group members, their distance apart, and their body orientations. These in turn are strongly influenced by the shape and size of the room in which a small group session is conducted and the arrangement of chairs and tables. A long, narrow room will probably limit eye contact along its length and impel members to talk to others across the room. Elongated tables may create a physical barrier which may be reassuring in groups where formality is of the essence, or where there is a wish to maintain personal distance or space. Personal space, the area around a person which he or she regards as private, will of course vary for an individual, and some students prefer to sit on a little distance between them and others. Moreover, while making seating arrangements one should be aware that small group room should be noise free, everyone must be equally spaced, tutor likely to have a central position and everyone may be able to make eye contact with each other. It should be kept in mind that the in small group sessions the room environment should be according to the weather not too warm or cool, relax or tense, free or constrain, harmonious or dissonant.^13^

***[viii] Small group teaching environment:*** While establishing a small group, it should be kept in mind that the small-group environment must be designed to promote cooperation, rather than competition, to facilitate trust for working together to learn. In order to achieve a better small group teaching and learning environment, it is important to develop appropriate groups so that students can be actively involved in the activities.^14^ The faculty has an important role in providing a pleasant environment in which the learners learn happily. Pleasant academic environment promote a culture through knowing and valuing each student and respect for individuals. A constructive environment can not only release more energy and imagination in a group, but affects the way students feel about belonging to it.

***[ix] Resources:*** The role of the faculty is also to ensure that adequate resource material including books, articles, e-learning, simulated or real patients are available to facilitate the teaching and learning as per requirement. It has also been suggested that in teaching and learning sessions tutor should facilitate the students that they may present their discussion on PowerPoint as well on chalk board, as combined tool is preferable to achieve the better knowledge.^15^^,^^16^

***[x] Preparation of the session:*** It is worth remembering that students' confidence, communication skills vary according to their experience and environment. Therefore, the tutor should assign an appropriate topic to the students. Provide them with a brief agenda with a question for which they might think about and allocate specific reading to ensure the topic is kept in focus. A plan should be developed to promote self-confidence and willingness among the students to take risks through structured support, valuing the effort and recognition of their work. It must be suggested that all the students should prepare, not just the presenter.^8^ Moreover, provide all the required resources including books, articles, chalkboard and PowerPoint facilities.

***[xi] Diagnosis of the needs:*** The assessment of needs from all the identified stakeholders is necessary step for the successful running of small group teaching and learning sessions. Input from the students, teachers, administrators and support staff may ensure that the resultant session may be responsive and reflective of the needs of the concerned students. This step helps to identify the scope of the administrative and organizational impact of the session. The assessment of the needs can be used to help the committee in decision-making such as setting the goals and objectives in the physiology.

***[xii] Formulation of the objectives:*** The instructor should think about what objectives he / she is trying to achieve; what he / she wants from the students to have learned from the contents of small group discussion. Before assigning a small group session the tutor should know about what the students already know; their level of experience; degree of interest and difficulty of the topic; the ways they learn new material and such practical issues as the length of the small group session. While formulating the objectives, the committee members should decide on educational purpose to be attained in that specific session. The faculty may derive these objectives from systematic studies of the learners, and from the analysis of the subject matter. A plan may be developed to effectively organize the educational experiences and observe whether the educational purposes would be attained or not. Objective evaluation instruments (e.g., tests, work samples, questionnaires, and records) may develop to check the effectiveness of the small group sessions. The members of small group teaching committee need to coordinate with the curriculum committee to consider the contents requirement enabling the student to achieve the objectives or outcomes and which contents of the subject will be included or excluded should be based on the decision of the committee. It is also suggested to select the more applied contents for small group teaching such as in respiratory Physiology applications of the lung function / Spirometry.

***[xiii] Small group teaching outline:*** In the present review, proving an example of sessions in the lung function lab. The small group teaching and learning session should be based on developing a discussion to enhance learning in the lung function lab. The components of the small group teaching will be based on the introduction to Spirometry, parameters, international standards, purpose of Spirometry, indications, contraindications, quality assurance, equipment calibration, infection control, test performance, acceptability, reproducibility criteria, predicted and measured reference, normal range, equations, interpretation, diagnostic pattern and severity of the disease.

***[xiv] Group maintenance role:*** This requires the ability to observe what is happening in the small group in order to ensure the group functions are going well. The facilitator must observe what is happening, are the group members participating equally, are anyone dominating, is anyone undermining the group process, who holds the power, resolve the conflicts when they arise.

***[xv] The task role:*** The facilitator must ensure that the group completes the task, achieves the stated outcomes for the session. Once the group members are clear about the expectations, the facilitator must ensure that they remain focused on the task, explain the task, question understanding, keep time, clarify and summarize progress, close the session. The facilitator may also provide appropriate stimulus material and resource so that the group can achieve the task set.^15^^-^^17^

**Table-III:** The role of instructor during the small group session

Facilitate small group teaching and learning sessions
Prepare the session based on current trends in science / research oriented ideas
Develop eye contact to all the students of the session
Understand how to handle the intelligent and poor students
Frequently ask the questions / provide chance to students they ask the questions and enhance the discussion
Pay attention and reply friendly till students fully understand
Reply the answer in simple words and give easy and appropriate examples
Provide positive feedback and avoid criticism even the student unable to give answer^18^
Conclude the session with a simple and significant take home message

***[xvi] Getting unstuck:*** If you wish to avoid getting stuck try to avoid questions which suggest one answer that can be based on yes or no. Try to ask questions which give informative answers. Avoid verbal checklists, ask questions directed at higher levels of objectives. Once you have asked a question wait for an answer. Short silence is not a wrong, they often get students talking more freely in the long run once they know you require and appreciate an honest answer to an honest question.

***[xvii] Ending of the session:*** Debriefing the group on the activity is essential because it maximize the learning. Debriefing summarizes, clarifies what has been learnt.^9^ After the small group activity, evaluate the successfulness of the session by asking that did you understand the objectives of the topic, are you motivated to learn. Moreover, feedback must be given as it enhances the awareness of strength and areas for improvement, and identifies the actions to be taken to improve the performance.^18^ Moreover, reflect on the experience by getting feedback that how it can be improved next time. The completing of discussion in a positive way is important, summarizing the main points can help and leading into the next topic reinforces direction and provides momentum. At the end of the session appreciate all the students for their active participation.

## CONCLUSION

Small-group teaching and learning sessions provide productive academic environment, strategy for dynamic and collaborative learning both in basic and clinical medical science. Small group teaching offers active participation of learners, increase the teamwork ability, retention of knowledge, enhance transfer of concepts to new problems, increase student interest, and improve the self-directed learning and critical skills. It develops self-motivation, exploration of issues, deep learning, higher-order activities and enhances student-faculty and peer-peer interaction and improves the communication skills.

## Notes

***Statement of proprietary interest:*** No conflict of interest with any institution / organization.
